# Curricular Approach to IPE: Preparing Health Professions Students to Deliver Team-Based Care

**DOI:** 10.1093/geroni/igab046.404

**Published:** 2021-12-17

**Authors:** Stacy Barnes, Kelly Horton

**Affiliations:** Marquette University, Milwaukee, Wisconsin, United States

## Abstract

Interprofessional education (IPE) is essential to prepare students for future healthcare careers and to meet accreditation requirements for health profession schools. After surveying successful IPE programs across the country, Marquette University developed a curricular approach. Over 1,500 students from 10 health professions (Athletic Training, Medical Laboratory Science, Counseling Psychology, Dentistry, Medicine, Nursing, Occupational Therapy, Physical Therapy, Physician Assistant Studies, Speech-Language Pathology) currently participate in a series of four interactive, half-day courses which are aligned with the Interprofessional Education Collaborative (IPEC) core competencies. Courses were moved online in response to the pandemic and are currently delivered using Microsoft Teams. Feedback from learners and faculty is gathered using post-event surveys and has been overwhelmingly positive. Learner outcomes are measured using the Interprofessional Collaborative Competencies Attainment Survey. Overall, this approach has proven to be an effective and efficient model for delivering IPE to large numbers of students.

